# Incident heart failure in chronic kidney disease: proteomics informs biology and risk stratification

**DOI:** 10.1093/eurheartj/ehae288

**Published:** 2024-05-17

**Authors:** Ruth F Dubin, Rajat Deo, Yue Ren, Jianqiao Wang, Alexander R Pico, Josyf C Mychaleckyj, Julia Kozlitina, Victoria Arthur, Hongzhe Lee, Amil Shah, Harold Feldman, Nisha Bansal, Leila Zelnick, Panduranga Rao, Nidhi Sukul, Dominic S Raj, Rupal Mehta, Sylvia E Rosas, Zeenat Bhat, Matthew R Weir, Jiang He, Jing Chen, Mayank Kansal, Paul L Kimmel, Vasan S Ramachandran, Sushrut S Waikar, Mark R Segal, Peter Ganz, CRIC Study Investigators, Lawrence J Appel, Debbie L Cohen, James P Lash, Robert G Nelson, Vallabh O Shah, Mark L Unruh

**Affiliations:** Division of Nephrology, University of Texas Southwestern Medical Center, 5323 Harry Hines Blvd, H5.122E, Dallas, TX 75390, USA; Division of Cardiovascular Medicine, Perelman School of Medicine at the University of Pennsylvania, Philadelphia, PA, USA; Department of Biostatistics, Epidemiology, and Informatics, Perelman School of Medicine, University of Pennsylvania, Philadelphia, PA, USA; Harvard T.H. Chan School of Public Health, Boston, MA, USA; Institute of Data Science and Biotechnology, Gladstone Institutes, San Francisco, CA, USA; Center for Public Health Genomics, University of Virginia School of Medicine, Charlottesville, VA, USA; McDermott Center for Human Growth and Development, University of Texas Southwestern Medical Center, Dallas, TX, USA; Division of Cardiology, University of Texas Southwestern Medical Center, Dallas, TX, USA; Department of Biostatistics, Epidemiology, and Informatics, Perelman School of Medicine, University of Pennsylvania, Philadelphia, PA, USA; Division of Cardiology, University of Texas Southwestern Medical Center, Dallas, TX, USA; Patient-Centered Outcomes Research Institute, Washington, DC, USA; Division of Nephrology, University of Washington Medical Center, Seattle, WA, USA; Division of Nephrology, University of Washington Medical Center, Seattle, WA, USA; Division of Nephrology, University of Michigan, Ann Arbor, MI, USA; Division of Nephrology, University of Michigan, Ann Arbor, MI, USA; Division of Kidney Diseases and Hypertension, George Washington University School of Medicine, Washington, DC, USA; Division of Nephrology and Hypertension, Northwestern University Feinberg School of Medicine, USA; Joslin Diabetes Center, Beth Israel Deaconess Medical Center, Harvard Medical School, Boston, MA, USA; Division of Nephrology, University of Michigan, Ann Arbor, MI, USA; Division of Nephrology, Department of Medicine, University of Maryland School of Medicine, Baltimore, MD, USA; Department of Epidemiology, Tulane University, New Orleans, LA, USA; Department of Epidemiology, Tulane University, New Orleans, LA, USA; Division of Cardiology, University of Illinois College of Medicine, Chicago, IL, USA; Division of Kidney, Urologic, and Hematologic Diseases, National Institute of Diabetes and Digestive and Kidney Diseases, National Institutes of Health, Bethesda, MD, USA; University of Texas School of Public Health San Antonio and the University of Texas Health Sciences Center in San Antonio, Section of Preventive Medicine and Epidemiology, Department of Medicine, Boston University Chobanian & Avedisian School of Medicine, Boston, MA, USA; Section of Nephrology, Department of Medicine, Boston University Chobanian & Avedisian School of Medicine, Boston, MA, USA; Department of Epidemiology and Biostatistics, University of California San Francisco, San Francisco, CA, USA; Division of Cardiology, University of California San Francisco, San Francisco, CA, USA

**Keywords:** Heart failure, Chronic kidney disease, Risk model, Mendelian randomization

## Abstract

**Background and Aims:**

Incident heart failure (HF) among individuals with chronic kidney disease (CKD) incurs hospitalizations that burden patients and health care systems. There are few preventative therapies, and the Pooled Cohort equations to Prevent Heart Failure (PCP-HF) perform poorly in the setting of CKD. New drug targets and better risk stratification are urgently needed.

**Methods:**

In this analysis of incident HF, SomaScan V4.0 (4638 proteins) was analysed in 2906 participants of the Chronic Renal Insufficiency Cohort (CRIC) with validation in the Atherosclerosis Risk in Communities (ARIC) study. The primary outcome was 14-year incident HF (390 events); secondary outcomes included 4-year HF (183 events), HF with reduced ejection fraction (137 events), and HF with preserved ejection fraction (165 events). Mendelian randomization and Gene Ontology were applied to examine causality and pathways. The performance of novel multi-protein risk models was compared to the PCP-HF risk score.

**Results:**

Over 200 proteins were associated with incident HF after adjustment for estimated glomerular filtration rate at *P* < 1 × 10^−5^. After adjustment for covariates including N-terminal pro-B-type natriuretic peptide, 17 proteins remained associated at *P* < 1 × 10^−5^. Mendelian randomization associations were found for six proteins, of which four are druggable targets: FCG2B, IGFBP3, CAH6, and ASGR1. For the primary outcome, the *C*-statistic (95% confidence interval [CI]) for the 48-protein model in CRIC was 0.790 (0.735, 0.844) vs. 0.703 (0.644, 0.762) for the PCP-HF model (*P* = .001). *C*-statistic (95% CI) for the protein model in ARIC was 0.747 (0.707, 0.787).

**Conclusions:**

Large-scale proteomics reveal novel circulating protein biomarkers and potential mediators of HF in CKD. Proteomic risk models improve upon the PCP-HF risk score in this population.

## Structured Graphical Abstract

**Structured Graphical Abstract:**
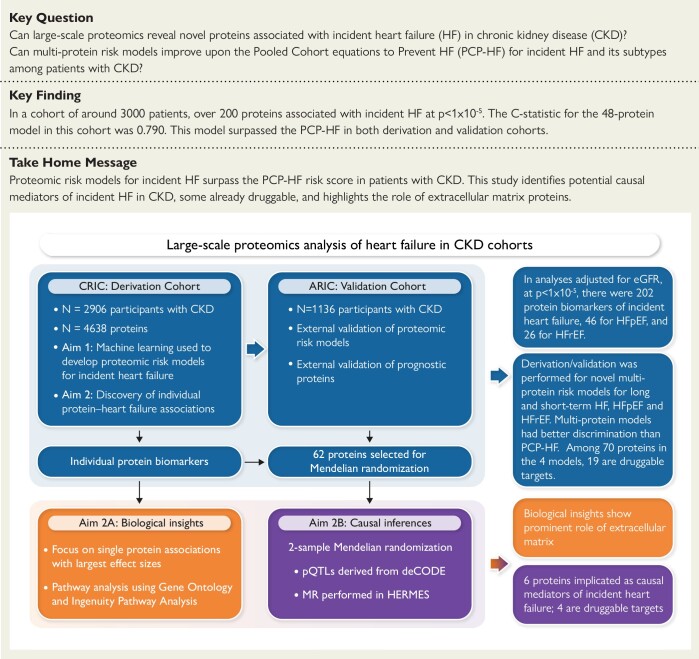
Large-scale proteomics analysis of heart failure in CKD cohorts. CKD, chronic kidney disease; PCP-HF, Pooled Cohort equations to Prevent Heart Failure; CRIC, Chronic Renal Insufficiency Cohort; ARIC, Atherosclerosis Risk in Communities; HERMES, Heart Failure Molecular Epidemiology for Therapeutic Targets Consortium; MR, Mendelian randomization; eGFR, estimated glomerular filtration rate; HF, heart failure; HFpEF, heart failure with preserved ejection fraction; HFrEF, heart failure with reduced ejection fraction; pQTL, protein quantitative trait loci.

**See the editorial comment for this article ‘Proteomics for understanding progression to heart failure in chronic kidney disease: promising but still not there’, by F. Zannad and J.P. Ferreira, https://doi.org/10.1093/eurheartj/ehae399.**

## Introduction

The annual Medicare cost of cardiovascular hospitalizations among patients with chronic kidney disease (CKD) exceeded $5 billion in 2018. Compared to populations without CKD, heart failure (HF) is more common in patients with CKD, affecting 28% of Medicare recipients over the age of 66 with CKD, compared to 6% of their counterparts without CKD.^1^ Thus, there is an urgent need to elucidate biological mechanisms underlying HF pathogenesis and to identify new therapeutic targets for preventing incident HF among these patients. In addition to understanding the underlying biology, better risk stratification for incident HF in CKD is needed.

Proteins regulate biological processes and reflect not only genetic programming but also the influence of environment, age, comorbidities, lifestyle, and medications.^2–4^ Multi-protein models predict the risk of diseases and clinical outcomes as well as or better than traditional clinical models^2–4^ or genetic scores.^5^ Moreover, such protein models consist of modifiable biological factors and could be used to monitor responses to medical or lifestyle interventions. For example, a prognostic equation for cardiovascular risk that consisted of modifiable protein risk factors accurately predicted which patients remained at high risk for poor outcomes and might benefit from more specialized therapies.^6^ The Chronic Renal Insufficiency Cohort (CRIC) is an exceptionally well-phenotyped resource for studying incident HF in CKD. Participants have estimated glomerular filtration rate (eGFR) values calibrated to iothalamate-measured GFR. There are physician-adjudicated HF and HF subtypes [HF with preserved ejection fraction (HFpEF) and HF with reduced ejection fraction (HFrEF)], extensive medical information, and proteinuria. In the present investigation, we utilized SomaScan V.4.0 (SomaLogic, Boulder, CO), a large-scale aptamer proteomic platform that simultaneously measures nearly 5000 distinct plasma proteins. We applied SomaScan to study proteins associated with the primary outcome of 14-year HF and the secondary outcomes of 4-year HF, 14-year HFpEF, and 14-year HFrEF in CRIC and validated our findings in the Atherosclerosis Risk in Communities (ARIC) study.

We constructed our study of incident HF and HF subtypes (HFpEF and HFrEF) in patients with CKD around several aims. We sought to: (i) discover and validate novel circulating proteins associated with HF as biomarkers and potential mediators of the primary and secondary outcomes; (ii) define and prioritize biological pathways associated with incident HF; (iii) identify proteins that may be causal mediators of incident HF through Mendelian randomization (MR) analyses; and (iv) construct multi-protein prognostic models for incident HF in the CKD population and assess if they have better prognostic utility and are more modifiable than clinical risk equations for incident HF developed in the general population, such as the Pooled Cohort equations to Prevent HF (PCP-HF) risk score.^7^

## Methods

### Participants

Our derivation cohort included CRIC participants at Visit 5 (*n* = 3417) who had blood samples assayed for proteomics; Visit 5 occurred 1 year following the CRIC first baseline visit. We excluded those with poor sample quality (*n* = 105), kidney failure on dialysis (*n* = 51), and systemic lupus erythematosus (*n* = 12) (lupus antibodies are thought to interact with the aptamer reagents). We also excluded those with history of HF at baseline (*n* = 284) or Visit 5 (*n* = 47), or those with no cystatin C or creatinine measured (*n* = 12). The remaining 2906 participants constituted our analytical sample. External validation of the CRIC findings was performed in 1136 participants of the ARIC study with eGFR < 60 mL/min/1.73 m^2^ and proteomics assays at Visit 5.

### Predictors

#### Proteins

Circulating proteins were measured with the SomaScan V4.0 platform, which includes 5284 aptamers. We excluded 305 aptamers paired with non-human proteins, 130 aptamers under investigation, and 19 aptamers with >50% coefficient of variation (CV) in SomaLogic’s quality control report. After these exclusions, 4830 aptamers and 4638 unique proteins remained (some proteins are measured by 2 or more aptamers) (see Supplementary data online, *Methods* and Supplementary data online, *Table S1* for additional quality control information).

### Incident heart failure outcomes

We designated the primary outcome as incidence of 14-year HF, the maximum follow-up currently available. We examined 4-year HF, 14-year HFpEF, and 14-year HFrEF as secondary outcomes. The short-term 4-year time horizon was chosen to identify patients at particularly high risk of near-term HF events, consistent with NHLBI workshop recommendations^8^ and also used in our prior studies.^2^ Fourteen-year horizons were chosen for HF subtypes in order to maximize statistical power. Preserved ejection fraction (EF) was defined as EF ≥ 50% (see Supplementary data online, *Methods* for HF adjudication in CRIC).

### Statistical analysis

Summary statistics for the CRIC participants’ baseline characteristics were calculated as mean and standard deviation (SD) for symmetric variables and median and interquartile range (IQR) for skewed variables. SomaLogic normalizes the entire protein dataset using adaptive normalization by maximum Likelihood (ANML) to remove unwanted biases in the assay. To place proteins on the same scale, we normalized using median absolute deviation (MAD) units. We ranked protein associations with study outcomes using HR per MAD unit. In presentation tables, we show HR per doubling of protein using log2 transformation instead of MAD, since log2 is a more common effect size unit in epidemiology. Individual protein associations with primary and secondary HF outcomes were modelled with Cox regression. This is primarily a discovery project of potential new protein markers, with a reasonable expectation of multiple true associations. For this reason, the primary threshold considered for individual protein–HF associations was false discovery rate (FDR) < 0.05, as this cut-off reflected the use of multiple tests but permitted less type II error than Bonferroni correction. We also provide the Bonferroni corrected *P*-value for individual protein association, and for ARIC validation, to highlight the strongest associations. *C*-statistics are compared by *t*-test using a one-sided *P*-value (survcomp::cindex.comp). See Supplementary data online, *Methods* for additional information on CRIC participants, protein assay quality control, clinical and laboratory variables, HF adjudications, multivariable models, interaction testing, functional enrichment with Gene Ontology (GO) and Ingenuity Pathway Analysis, two-sample MR utilizing the deCODE and HERMES databases, and external validation of our protein findings in ARIC.

### Derivation of risk models in CRIC

We developed protein, clinical, and hybrid clinical-protein risk models for the primary outcome (14-year HF) and secondary outcomes (4-year HF, 14-year HFrEF, and 14-year HFpEF). For all models developed in this project, we randomly split the CRIC data into two sets: an 80% training set and 20% testing set. The training set was used to build the predictive models, and the testing set was used only to evaluate the model performance. We found no violations of proportional hazard assumptions for clinical and protein models.

#### Clinical models

We examined the following clinical models for HF: (i) original PCP-HF with published coefficients in four subgroups of gender and race;^7^ (ii) PCP-HF, refit to CRIC; (iii) refit PCP-HF + eGFR + proteinuria; and (iv) a novel clinical HF model developed in the CRIC population. Separately, we developed a new clinical model from a list of 29 clinical variables, including PCP-HF and also CKD-specific factors, shown in Supplementary data online, *Table S2*. The base clinical model was developed using backward Cox regression from a list of clinical variables that had associations with the outcome at *P* < .05, after adjustment for age, gender, and eGFR. Variables with the greatest *P*-value were removed from the model until all remaining variables had a significant *P*-value < .1. The base clinical model did not include NT-proBNP, left ventricular mass index (LVMI), or EF, but these were added as continuous variables to subsequent expanded clinical models.

#### Protein-only and clinical-protein hybrid models

Our frontline technique for developing protein and hybrid models was elastic-net (EN) Cox regression which combines ridge (L2) and LASSO (L1) penalties and handles time-to-event outcomes. Model fitting was conducted using the R package *glmnet*. The relative contributions of the two penalties are controlled by a mixing parameter α which we set to 0.5 for balance. The shrinkage (regularization) parameter γ which controls model complexity (the number of included proteins) was determined by 10-fold cross validation and the ‘1 standard error rule’. After arriving at the final selection of protein and/or clinical factors (hybrid models), to reduce bias in estimated regression coefficients, we refit the selected features for the EN model in a Cox regression model.^9^ For protein-only, and protein-clinical hybrid models, N-terminal pro-B-type natriuretic peptide (NT-proBNP) was included in the set of candidate risk factors; for protein-clinical-echo hybrid models, NT-proBNP, LVMI, and EF as continuous variables were included among candidate risk factors. Analyses were performed using R version 4.2.1. Additional methods for evaluating risk models are found in Supplementary data online, *Methods*.

## Results

### CRIC derivation cohort and heart failure outcomes

Among the 2906 CRIC participants, the median (IQR) age was 59.3 (11) years, eGFR was 42.1 (30.9, 54.4) mL/min/1.73 m^2^, 40% were Black individuals, 46% had diabetes mellitus (DM), and 18% had a history of myocardial infarction (MI). Those with incident HF (*P* < .01) and also those who developed HFpEF as opposed to HFrEF (*P* = .04) had lower eGFR (*Table 1*). There were a total of 390 (13%) incident HF events over a total of 14 years of follow-up (median [IQR] 9.2 [4, 12.4 years]). Of these, 137 events were HFrEF and 165 were HFpEF. There were 183 (6.3%) HF events over 4 years. At the time of the incident HF event, median [IQR] EF was 37 [25, 43] and 59 [55, 65] for individuals with HFrEF and HFpEF, respectively.

**Table 1:** Characteristics of CRIC participants stratified by heart failure outcome

	All participants without HF at the study baseline	Participants without incident HF during follow-up	Participants with incident HF during follow-up				
		No HF	Any HF	*P*-value No HF vs. any HF	HFrEF	HFpEF	*P*-value HFpEF vs. HFrEF
*n*	2906	2516	390		137	165	
Age (years)	61.2 [53.6, 67.2]	60.6 [52.5, 66.5]	64.4 [58.7, 70.5]	<.01	65.4 [61.2, 71.7]	63.5 [58.2, 69.9]	.06
Men	1613 (55.5)	1389 (55.2)	224 (57.4)	.44	89 (65.0)	89 (53.9)	.07
Black race	1144 (39.4)	975 (38.8)	169 (43.3)	.10	53 (38.7)	75 (45.5)	.29
Hispanic ethnicity	320 (11.0)	281 (11.2)	39 (10.0)	.55	11 (8.0)	16 (9.7)	.76
Diabetes	1347 (46.4)	1089 (43.3)	258 (66.2)	<.01	84 (61.3)	117 (70.9)	.10
Hypertension	2557 (88.1)	2184 (86.9)	373 (95.6)	<.01	132 (96.4)	158 (95.8)	1.00
Hypertension treatment	2636 (90.7)	2257 (89.7)	379 (97.2)	<.01	134 (97.8)	160 (97.0)	.93
Diabetes medication (%)	749 (25.9)	610 (24.4)	139 (35.7)	<.01	55 (40.1)	58 (35.2)	.44
Myocardial infarction (%)	536 (18.4)	388 (15.4)	148 (37.9)	<.01	63 (46.0)	55 (33.3)	.03
Stroke	280 (9.6)	216 (8.6)	64 (16.4)	<.01	18 (13.1)	31 (18.8)	.24
Atrial fibrillation	432 (14.9)	329 (13.1)	103 (26.4)	<.01	39 (28.5)	42 (25.5)	.65
Peripheral vascular disease	184 (6.3)	139 (5.5)	45 (11.5)	<.01	15 (10.9)	19 (11.5)	1.00
Systolic blood pressure (mmHg)	122.7 [112.0, 138.0]	122.7 [110.7, 136.7]	128.7 [117.3, 144.0]	<.01	127.3 [114.0, 142.0]	130.0 [116.7, 146.7]	.27
Diastolic blood pressure (mmHg)	69.3 [61.3, 78.0]	69.3 [61.3, 78.7]	66.0 [58.7, 76.0]	<.01	67.0 [59.3, 78.8]	66.0 [58.7, 74.7]	.22
Heart rate	66.0 [60.0, 76.0]	66.0 [60.0, 76.0]	66.0 [60.0, 76.0]	.44	66.0 [60.0, 74.0]	66.0 [60.0, 76.0]	.75
Current smoking	351 (12.1)	299 (11.9)	52 (13.3)	.46	17 (12.4)	24 (14.5)	.71
Body mass index (kg/m^2^)	30.6 [26.6, 35.9]	30.3 [26.5, 35.3]	33.2 [28.8, 38.9]	<.01	32.4 [28.7, 38.0]	33.3 [29.2, 39.1]	.28
eGFR (mL/min/1.73 m^2^)	42.1 [30.9, 54.4]	43.3 [31.6, 55.5]	36.8 [27.2, 48.1]	<.01	38.0 [27.6, 48.9]	33.5 [24.9, 44.4]	.04
CKD stage (%)				<.01			.53
CKD 1	14 (0.5)	13 (0.5)	1 (0.3)		0 (0.0)	1 (0.6)	
CKD 2	494 (17.0)	469 (18.6)	25 (6.4)		9 (6.6)	9 (5.5)	
CKD 3A	758 (26.1)	666 (26.5)	92 (23.6)		35 (25.5)	30 (18.2)	
CKD 3B	963 (33.1)	810 (32.2)	153 (39.2)		54 (39.4)	66 (40.0)	
CKD 4	619 (21.3)	506 (20.1)	113 (29.0)		37 (27.0)	56 (33.9)	
CKD 5	58 (2.0)	52 (2.1)	6 (1.5)		2 (1.5)	3 (1.8)	
Urine protein (g/day)	0.2 [0.1, 0.8]	0.1 [0.1, 0.8]	0.2 [0.1, 1.1]	<.01	0.2 [0.1, 0.9]	0.3 [0.1, 1.4]	.15
HDL (mg/dL)	46.0 [38.0, 57.0]	46.0 [38.0, 57.0]	44.0 [36.0, 54.2]	<.01	44.0 [36.0, 52.8]	44.0 [35.0, 54.0]	.94
LDL (mg/dL)	96.0 [76.0, 120.0]	98.0 [77.0, 120.0]	88.5 [71.0, 114.0]	<.01	89.0 [70.2, 106.8]	88.5 [74.0, 119.2]	.26
Triglycerides (mg/dL)	125.0 [89.0, 182.0]	125.0 [88.0, 182.0]	129.0 [89.8, 185.2]	.42	132.0 [89.2, 182.8]	131.5 [100.0, 196.0]	.34
Total cholesterol (mg/dL)	178.0 [153.5, 207.0]	180.0 [155.0, 208.0]	168.0 [145.8, 198.0]	<.01	164.0 [146.0, 195.8]	169.5 [149.0, 202.2]	.14
Serum albumin (g/dL)	4.1 [3.8, 4.3]	4.1 [3.8, 4.4]	4.0 [3.7, 4.3]	<.01	4.1 [3.8, 4.3]	4.0 [3.7, 4.3]	.07
Haemoglobin (g/dL)	12.9 (1.8)	13.0 (1.8)	12.4 (1.8)	<.01	12.5 (1.6)	12.3 (1.9)	.21
Fasting glucose (mg/dL)	98.0 [88.0, 121.0]	97.0 [87.0, 117.0]	108.0 [92.0, 143.5]	<.01	107.0 [91.5, 133.0]	114.0 [93.0, 153.0]	.34
QRS interval (ms)	92.0 [86.0, 100.0]	92.0 [84.0, 100.0]	96.0 [88.0, 108.0]	<.01	98.0 [92.0, 114.0]	94.0 [86.0, 102.0]	<.01
Left ventricular mass index (g/m^2.7^)	61.6 [49.8, 71.2]	59.9 [48.3, 69.5]	69.7 [61.8, 85.3]	<.01	71.5 [62.5, 90.8]	69.2 [60.3, 82.7]	.11
Ejection fraction (%)	55.5 [52.7, 58.5]	55.6 [53.1, 58.7]	54.3 [50.3, 56.6]	<.01	52.0 [43.2, 56.0]	54.5 [51.9, 56.8]	<.01

Baseline characteristics are described as *n* (%), median [IQR], or mean (SD). *P*-values are calculated as *t*-test or χ^2^. NT-proBNP and parathyroid hormone were measured as aptamers in the SomaScan platform, with units of relative fluorescent units.

### ARIC validation cohort and heart failure outcomes

Compared to the CRIC participants, the preselected subset of 1136 ARIC participants were older, had higher eGFR, fewer were Black, and fewer had a history of cardiovascular disease (CVD). The mean (SD) age was 77.4 (5.4) years, the median (IQR) eGFR was 51.06 [44, 56.1] mL/min/1.73 m^2^, 16% were Black, and 6% had a history of MI (see Supplementary data online, *Table S3*). Compared to CRIC participants, there were fewer ARIC participants in CKD stages 2 and 4: in CRIC, 18% had eGFR ≥ 60 (CKD stage 2), vs. 0% in ARIC; in CRIC, 23% had eGFR 15–30 (CKD stage 4), vs. 5% in ARIC. There were 149 (13%) incident HF events over a total of 9 years of follow-up (median [IQR] 7 [5.0, 7.7] years), of which 52 events were HFrEF and 76 HFpEF. There were 72 HF events (6.3%) over 4 years.

### Associations of individual clinical risk factors with heart failure in CRIC

Traditional risk factors observed in CRIC participants who developed incident HF (either HFrEF or HFpEF) were older age, DM, hypertension (HTN), history of CVD or peripheral vascular disease, higher systolic blood pressure (SBP), higher body mass index (BMI), longer QRS interval, and higher LVMI.^7,10^ Chronic kidney disease-related risk factors for incident HF included lower eGFR, and higher urine protein to creatinine ratio, lower LDL, and higher parathyroid hormone. Among those with incident HF, few clinical risk factors differed between HFpEF vs. HFrEF, although those who developed HFpEF had lower baseline eGFR, and those who developed HFrEF were more likely to have history of MI and low baseline EF (*Table 1*).

### Associations of individual proteins with heart failure in CRIC and ARIC

#### Primary outcome (long-term heart failure)

**Figure 1:**
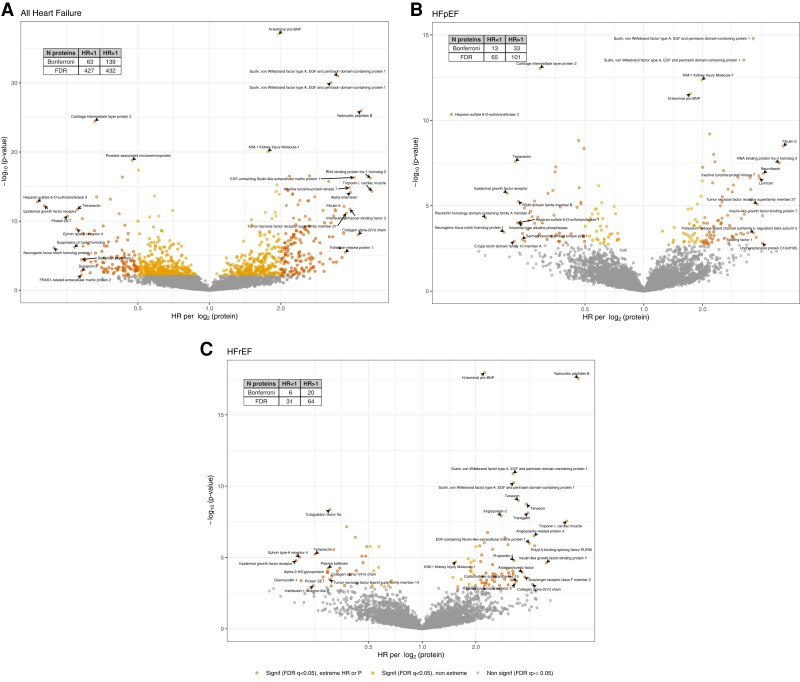
Individual proteins associated with heart failure after adjustment for eGFR in CRIC volcano plots of the associations of 4638 proteins with HF and HF subtypes, adjusted for eGFR. Extreme HR values are HR > 2 or <0.5

For the primary outcome of 14-year HF, among the 4638 proteins investigated, after adjustment for eGFR, there were 859 proteins (19% of all proteins measured) significant at FDR < 0.05 and 202 (4%) significant at *P* < 1 × 10^−5^ (*Figure 1*, Supplementary data online, *Table S4*). After adjustment for covariates including NT-proBNP, there were 81 proteins associated with HF at FDR < 0.05 and 17 unique proteins at *P* < 1 × 10^−5^ (see Supplementary data online, *Table S5*). Of these 17 unique proteins, three validated in ARIC, including NT-proBNP, angiopoietin-2^11,12^ and Sushi, von Willebrand factor type A, EGF and pentraxin domain-containing protein 1 (SVEP1)^13^ (*Table 2*). Overall, 16 proteins replicated for the association with general HF adjusted for eGFR, including ephrin type-A receptor 2,^20^ and also relatively novel proteins related to the extracellular matrix (ECM) such as WAP four-disulfide core domain protein 2^21^ and microfibril-associated glycoprotein 4^22^ (MFAP4) (*Table 2*). Eight of the 20 validated proteins in *Table 2* are druggable targets, as described in the Therapeutic Target Database.^28^

**Table 2:** Proteins associated with heart failure outcomes in both CRIC and ARIC

Protein	1) HF 2) HFrEF 3) HFpEF	HR^a^ per log2 in CRIC, adjusted for eGFR		HR^a^ per log2 in CRIC, full adjustment		Biological function	Relevant drug
		HR (95% CI)	*P*	HR (95% CI)	*P*		
Transgelin	1,3	3.18 (2.42,4.19)	1.87e^−16^	1.54 (1.10,2.17)	.012	Actin binding protein that regulates SMC contraction^14^ Knockout models have reduced LDL endocytosis.^15^ Up-regulated in arteriolar smooth muscle in PAH^16^	
N-terminal proBNP	1,2,3	1.96 (1.77,2.17)	8.5e^−38^	1.68 (1.49,1.90)	1.02e^−17^	Secreted by the ventricle in response to stretching due to volume overload	
EGF-containing fibulin-like extracellular matrix protein 1	1,3	4.07 (2.93,5.65)	4.9e^−17^	1.60 (1.08,2.37)	.019	Member of the fibulin family of ECM glycoprotein, modulates interactions between the basement membrane and ECM proteins. Stimulates MMP2, 9. Inhibits angiogenesis^17^ Binds and activates EGF receptor^18^	
Angiopoietin-2	1,3	2.11 (1.72,2.58)	5.42e^−13^	1.34 (1.08,1.65)	.0068	Endothelial destabilization^11^ Predicts incident heart failure, MESA^12^	LY3127804, trebananib
Sushi, von Willebrand factor type A, EGF and pentraxin domain-containing protein 1	1,3	3.48 (2.82,4.28)	1.02e^−31^	1.95 (1.52,2.52)	2.22e^−07^	SVEP1 Extracellular matrix protein. Found in epidemiological studies, human genetics, and animal studies, to contribute to atherosclerosis^13^	
Tumour necrosis factor receptor superfamily member 1A	1	2.27 (1.67,3.07)	1.41e^−07^	1.47 (1.03,2.10)	.033	Receptor can bind to and inhibit the cytotoxic activities of TNF alpha. Release of TNF1AR may act as a buffer against TNFalpha^19^	VB-111
Collagen alpha-1 (XXVIII) chain	1,3	3.25 (2.26,4.67)	1.81e^−10^	1.84 (1.19,2.86)	.0061	ECM component	Retinostat (targets endostatin, recombinant form)
Ephrin type-A receptor 2	1	2.64 (1.98,3.53)	5.39e^−11^	1.58 (1.12,2.22)	.0085	KO mice have more severe myocardial injury after ischemia^20^	CAR-T cells targeting EphA2
Growth/differentiation factor 15	1	2.18 (1.82,2.61)	3.12e^−17^	1.46 (1.17,1.83)	8.7 × 10^−4^	TGF-β protein. Contributes to endothelial dysfunction, atherosclerosis	
WAP four-disulfide core domain protein 2	1	2.22 (1.68,2.93)	2.28e^−08^	1.86 (1.34,2.60)	2.4 × 10^−4^	Protease inhibitor. AKA human epididymis protein 4 (HE4), may promote fibrosis by inhibiting serine proteases and MMPs, heart failure marker^21^	
Tumour necrosis factor ligand superfamily member 15	1	3.01 (2.21,4.10)	2.73e^−12^	1.93 (1.34,2.77)	4.2 × 10^−4^	AKA Vascular endothelial growth inhibitor (VEGI)	Vascular endothelial cell growth inhibitor (TNFSF15)
Microfibril-associated glycoprotein 4	1	2.12 (1.76,2.55)	2.34e^−15^	1.19 (0.96,1.47)	.108	ECM protein. Marker of hepatic fibrosis^22^	
Natriuretic peptides B	1,2	4.35 (3.32,5.69)	1.07e^−26^	2.78 (2.04,3.79)	1.24e^−10^	Secreted by the ventricle in response to stretching due to volume overload	
Macrophage scavenger receptor types I and II	1	2.14 (1.77,2.59)	3.77e^−15^	1.51 (1.23,1.85)	9.35e^−05^	Deposition of LDL in arterial walls leading to atherosclerotic plaque^23^	
Tyrosine-protein kinase transmembrane receptor ROR2	1,3	2.32 (1.76,3.06)	2.15e^−09^	1.72 (1.24,2.39)	.00123	Up-regulated in cardiac fibroblasts in remodelling hearts^24^	CCT301-38
Hepatitis A virus cellular receptor 2	1	2.33 (1.76,3.07)	2.74e^−09^	1.77 (1.31,2.41)	2.2 × 10^−4^	T cell immunoglobulin and mucin domain-containing protein 3 (Tim-3): T cell surface receptor that dampens immune response	MBG453
Calsyntenin-2	2	3.02 (1.93,4.73)^b^	1.27e^−06^	1.33 (0.82,2.16)^b^	.248	Regulates calcium-related synaptic transmission^25^	
Fibulin-5	3	5.19 (3.01,8.94)^c^	3.22e^−09^	3.25 (1.78,5.93)^c^	1.2 × 10^−4^	Secreted ECM protein found in large arteries;^26^ knockout mice have diastolic dysfunction^27^	
Collagen alpha-3 (VI) chain	3	2.28 (1.49,3.50)^c^	.000161	1.75 (1.05,2.90)^c^	.0304	ECM component	
Inactive tyrosine-protein kinase 7	3	3.16 (1.80,5.54)^c^	5.7e^−05^	2.46 (1.29,4.70)^c^	.0062	Wnt signalling	PF-06647020

Among proteins associated with the HF outcomes in CRIC after eGFR adjustment, at a significance of FDR < 0.05, we selected the 20 proteins with the highest HR and 10 with the lowest HR for the outcomes HF, HFpEF, and HFrEF, and sought to replicate these in ARIC. Shown above are the 20 proteins among 49 for which replication was attempted, that validated in ARIC for one or more of the three HF outcomes, after adjustment for eGFR, at *P* < .0017 (0.05/30). The fully adjusted model includes age, race, gender, DM, HTN, SBP, BMI, eGFR, UPCR, HDL, LDL, NT-proBNP, PTH, serum albumin, and haemoglobin. Druggable target search was performed using the Therapeutic Target Database, at http://db.idrblab.net/.

ECM, extracellular matrix; AKI, acute kidney injury; MMP, matrix metalloproteinases.

^a^HR is for the outcome 14-year general HF with the following exceptions.

^b^HFrEF.

^c^HFpEF.

#### Secondary outcomes (heart failure with reduced ejection fraction and heart failure with preserved ejection fraction)

For HFrEF, in analyses adjusted for eGFR, there were 95 proteins significant at FDR < 0.05 and 26 at *P* < 1 × 10^−5^ (*Figure 1*, Supplementary data online, *Table S6*). After full adjustment, two proteins remained for HFrEF (both natriuretic peptides) (see Supplementary data online, *Table S5*). Three proteins replicated in ARIC specifically for HFrEF in analyses adjusted for eGFR: calsyntenin-2,^25^ NT-proBNP, and natriuretic peptides B (*Table 2*). For HFpEF, in analyses adjusted for eGFR, there were 166 proteins at FDR < 0.05 and 46 at *P* < 1 × 10^−5^ (*Figure 1*, Supplementary data online, *Table S7*). After full adjustment, three remained at FDR < 0.05: NT-proBNP, macrophage metalloelastase, and SVEP1 (see Supplementary data online, *Table S5*). Ten proteins replicated in ARIC specifically for HFpEF, including relatively novel proteins such as fibulin-5 (FBLN5), transgelin,^14–16^ tyrosine-protein kinase transmembrane receptor ROR2 (ROR2), and inactive tyrosine-protein kinase 7.

To understand whether any individual protein associations differed between HFrEF and HFpEF, we examined proteins associated with HF at FDR < 0.05 with adjustment for eGFR, plotting hazard ratios (HRs) for HFrEF and HFpEF. A scatter plot of the effect sizes for HFpEF vs. HFrEF shows many HRs are similar and Spearman rho = 0.88 (see Supplementary data online, *Figure S1*). Formal interaction testing indicated that, among 846 proteins associated at FDR < 0.05 with 14-year HF after adjustment for eGFR, none had an interaction with EF < 50% that reached Bonferroni level significance. The 26 protein–HF associations for which there was an interaction at *P* < .05 are listed in Supplementary data online, *Table S8*, along with their associations with HFpEF and HFrEF. Additionally, we explored associations of individual proteins with HF with mildly reduced EF (HFmrEF) (EF 40%–49%; 43 events). Fourteen proteins associated with HFmrEF are listed in Supplementary data online, *Table S1*.

### Enrichment analysis of proteins associated with heart failure in CRIC

We carried out functional enrichment to examine biological processes associated with 14-year HF, HFrEF, and HFpEF. Our primary method was over-representation analysis using GO, comparing proteins associated with each outcome at FDR < 0.05 after adjustment for eGFR to the background of all proteins measured by the SomaScan assay. Significant GO terms included regulation of synapse assembly, proteoglycan biosynthetic process, cell adhesion, ECM organization, cellular response to hormone stimulus, and osteoblast differentiation. A review of the proteins within these diverse categories reveals the over-arching theme of vascular^29,30^ and myocardial ECM^31^ repair and composition, orchestrated by secreted glycoproteins, growth factors^32–34^ influencing ECM repair mechanisms, and receptors modulating the interaction between vascular smooth muscle and ECM (*Table 3*). Application of Gene Set Enrichment Analysis (GSEA) methodology to the GO database confirmed the prominence of ECM proteins for HF, HFpEF, and HFrEF and cell adhesion for HF and HFpEF. GSEA also highlighted the insulin receptor signalling pathway via phosphatidylinositol 3-kinase for HF (see Supplementary data online, *Table S9*). An alternate over-representation analysis using Ingenuity Pathway Analysis additionally showed prominence of the hepatic fibrosis pathway. Proteins in this pathway are collagen products and modulators that have roles in ECM remodelling, as well as inflammatory mediators including interleukins and tumour necrosis factor receptors^19^ (see Supplementary data online, *Table S10*).

**Table 3:** Gene Ontology terms associated with heart failure in CRIC

Gene Ontology term	1) HF 2) HFrEF 3) HFpEF	The ratio of gene hits to genes represented in SomaScan	*P*-value	Selected proteins within each GO term and their relevance to cardiovascular biology
Positive regulation of synapse assembly	1)	20/39	5.2 × 10^−6^	*Brain-derived neurotrophic factor and its receptor*: growth factor in cardiomyocytes, vascular smooth muscle, endothelium; influences vascular repair after injury.^29^*Thrombospondin-2* ECM protein: induces cardiac remodelling by activating MMPs, collagen production, TGF-β.^35^*Neurexin-1 and its receptor, Neuroligin-1* modulate cell to ECM adhesion in blood vessels.^30^
Proteoglycan biosynthetic process	1)	8/9	1.2 × 10^−5^	*Insulin-like growth factor I*: influences cardiac contractility, metabolism, hypertrophy, apoptosis.^36,37^*Glycosaminoglycan xylosylkinase, carbohydrate sulfotransferases 9, 11 and 12*: ECM composition.
Cell adhesion	1) 2) 3)	67/225 14/225 29/225	3.8 × 10^−5^ .00033 6.9 × 10^−9^	*Cadherin 8*: *cadherins facilitate vascular smooth muscle cell–ECM interaction*.^38^*Tenascin*: ECM protein. *Bone sialoprotein 2*: complexes with hydroxyapatite, forms the mineralized matrix. *Periostin*: facilitates cell–matrix crosstalk, contributes to myocardial fibrosis.^31^*Sushi, von Willebrand factor type A*: cell attachment process.
Extracellular matrix organization	2) 3)	13/122 16/122	1.8 × 10^−6^ 1.8 × 10^−5^	*MMP2, 12, 16; tenascin; periostin; Collagen alpha proteins; fibulin-5*: secreted ECM protein found in large arteries;^26^ knockout mice have diastolic dysfunction.^27^
Cellular response to hormone stimulus	2)	4/14	.00019	*Insulin-like growth factor-binding protein 7*: a potential marker for HFpEF.^33^*Slit homolog 2 protein*: secreted glycoprotein.
osteoblast differentiation	3)	16/53	4.8 × 10^−6^	*Insulin-like growth factors 2, 3, 5*: -2 contributes to atherosclerosis^34^ IGF system in CVD.^32^*Myocilin*: secreted glycoprotein, cell adhesion.

Over-representation analysis was performed using the Gene Ontology database for each 14-year HF outcome. We searched genes that corresponded to proteins having eGFR-adjusted associations with HF (*N* = 846 proteins), HFrEF (*N* = 88 proteins), or HFpEF (*N* = 160 proteins), at FDR < 0.05. GO terms listed here were found to be over-represented with statistical significance < 0.05 after Bonferroni correction for the number of terms.

ECM, extracellular matrix protein; OP, osteopontin; GDF, Growth differentiation factor; MMP, matrix metalloproteinase.

### Mendelian randomization

**Figure 2:**
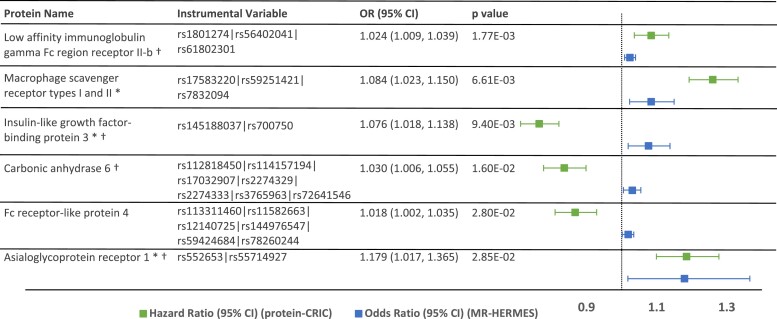
Mendelian randomization. Mendelian randomization (MR) in the HERMES genome-wide association study suggested causal associations for 6 proteins significant at *P*-value < .05 (corresponding to FDR < 0.2). pQTLs were identified in deCODE and the instrumental variable for each protein is shown above, along with the OR (95% CI) for MR. Three proteins with (*) replicated in ARIC at *P* < .05. Four proteins with (†) are potentially druggable targets (Therapeutic Target Database, at http://db.idrblab.net/)

Utilizing summary data from the Heart Failure Molecular Epidemiology for Therapeutic Targets (HERMES) Consortium genome-wide association study, two-sample MR suggested causal relationships for six proteins significant at FDR < 0.2 (corresponding to *P* < .05), including low affinity immunoglobulin gamma Fc region receptor II-b (FCG2B), insulin-like growth factor-binding protein 3 (IGFBP-3), macrophage scavenger receptor types I and II^23^ (MSRE), carbonic anhydrase (CAH6), Fc receptor-like protein 4 (FCRL4), and asialoglycoprotein receptor 1 (ASGR1) (*Figure 2*). Four of these proteins are identified as druggable targets using the Druggable Target Database:^28^ FCG2B, IGFBP3, CAH6, and ASGR1.

### Risk prediction models for heart failure in CRIC and ARIC

#### Clinical models

**Figure 3:**
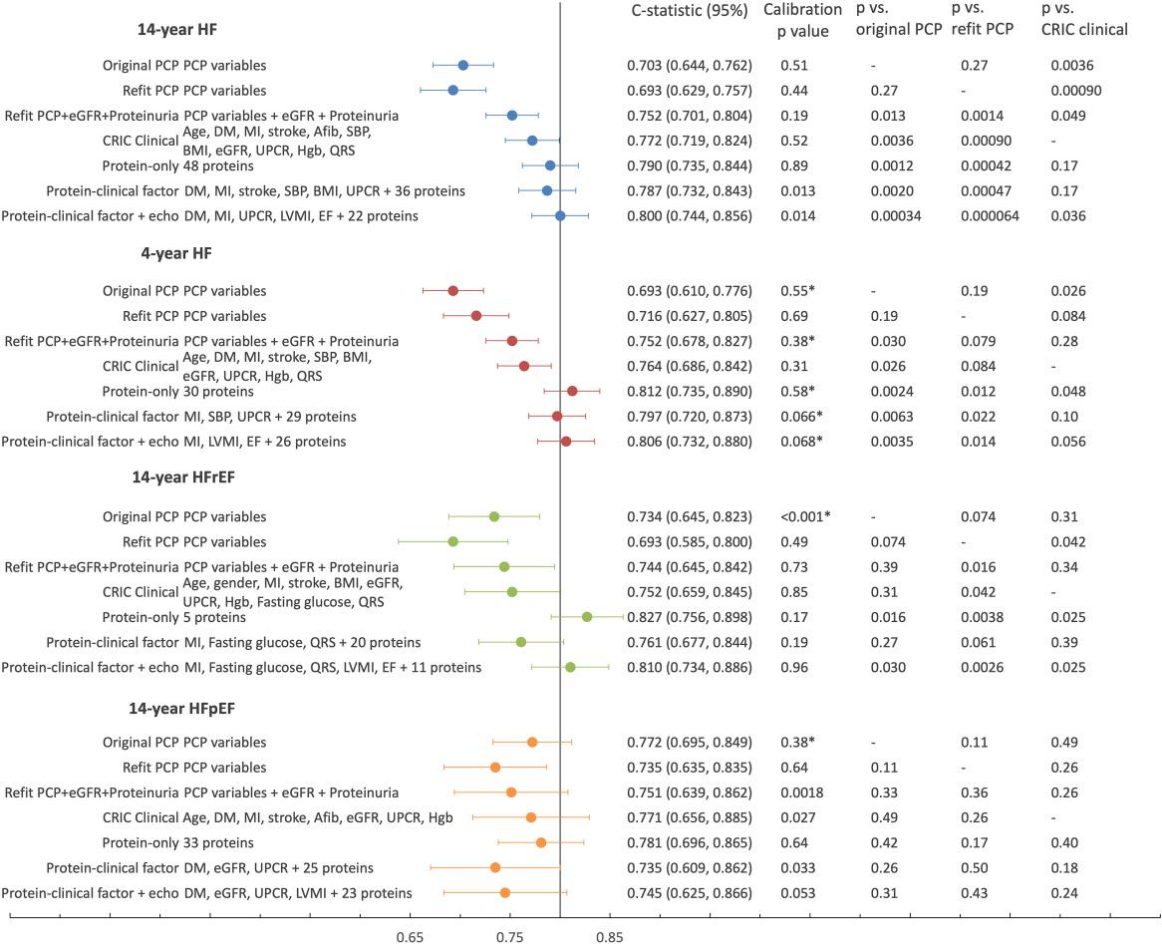
Clinical, protein, and hybrid risk models for incident heart failure in CRIC discrimination for risk models are shown in the 20% testing set (479 participants) and calibration in the 80% training set. In the testing set, there were 31 events for 4-year HF, 63 for 14-year HF, 23 for HFrEF, and 22 for HFpEF. Calibration is performed for quintiles of predicted risk, except for those marked with asterisk, which were analysed in quartiles. *C*-statistics are compared by *t*-test using a one-sided *P*-value. *C*-statistics for 14-year and 4-year HF PCP and protein models in gender- and race-specific subgroups are listed in Supplementary data online, *Table S11*

The original PCP-HF yielded similar *C*-statistics to the refit PCP-HF for 4-year and 14-year HF, but higher *C*-statistics for HFrEF and HFpEF (*Figure 3*; race and gender subgroups shown in Supplementary data online, *Table S11*). For subsequent comparisons in the 20% testing set, per our analysis plan, we focus on the refit PCP-HF.

The refit PCP-HF had moderate discrimination for the primary outcome of 14-year HF (*C*-statistic [95% CI] 0.693 [0.629, 0.757]) and HFrEF (0.693 [0.585, 0.800]), and good discrimination for 4-year HF (0.716 [0.627, 0.805]) and HFpEF (0.735 [0.635, 0.835]). The CRIC clinical models differed from the refit PCP model primarily in the inclusion of cardiovascular comorbidities such as MI, stroke, and atrial fibrillation, as well as CKD risk factors, eGFR, proteinuria, and haemoglobin (see Supplementary data online, *Table S12*). By design, the base CRIC clinical models excluded NT-proBNP, EF, and LVMI. Discrimination for the base CRIC clinical model was better than the refit PCP-HF for each outcome, particularly for the primary outcome of 14-year HF (*P* < .001) (*Figure 3*). Adding NT-proBNP, EF, and LVMI as continuous variables to the base clinical models improved discrimination: 14-year HF (*C*-statistic [95% CI] 0.802 [0.752, 0.852] vs. 0.772 [0.718, 0.825], *P* = .016), HFrEF (0.839 [0.773, 0.906] vs. 0.752 [0.658, 0.846], *P* = .001), and HFpEF (0.787 [0.674, 0.900] vs. 0.771 [0.656, 0.886], *P* = .15) (see Supplementary data online, *Table S13*).

#### Protein models

We developed separate multi-protein risk models for the primary outcome (14-year HF, 48 proteins) and secondary outcomes (4-year HF, 30 proteins; 14-year HFrEF, 5 proteins; 14-year HFpEF, 33 proteins). For the primary outcome of 14-year HF, discrimination of the 14-year HF protein model (0.790 [0.735, 0.844]) was significantly improved over the refit PCP (*P* < .001). Discrimination of the 4-year HF protein model (*C*-statistic 0.812 [0.735, 0.890]) and the 14-year HFrEF protein model (*C*-statistic 0.827 [0.756, 0.898]) was significantly better than the refit PCP or the base CRIC clinical model (*P* < .05 for all). Discrimination of the 14-year HFpEF protein model (0.781 [0.696, 0.865]) was numerically higher than the refit PCP (0.735 [0.635, 0.835]) but this difference did not reach statistical significance. Each protein model was well-calibrated (*Figure 3*). Individual proteins for each model, their beta-coefficients, associations with the outcome, and available drugs relevant to each protein are listed in Supplementary data online, *Tables S14*–*S17*. Druggable targets in each protein model numbered 13/48 for 14-year HF, 7/30 for 4-year HF, 1/5 for HFrEF, and 9/33 for HFpEF.^28^ Accounting for overlap, a total of 19 druggable targets were represented in the four protein models.

We developed hybrid clinical-protein models by applying EN to candidate risk factors that either included (i) proteins (including NT-proBNP) + clinical factors in the base CRIC clinical model, or (ii) proteins + clinical factors + EF, LVMI. Overall, these hybrid models showed discrimination that was similar to the protein model for each outcome. Discrimination of hybrid models for HFpEF was inferior to the protein model (*Figure 3*).

To provide additional comparison between risk models, we evaluated specificity, positive (PPV) and negative predictive values (NPV), and cases detected per 1000 participants per year. Complete results are tabulated in Supplementary data online, *Table S18* for 4-year and 14-year HF.

#### External validation of protein models in ARIC

In the validation for the proteomic models in 1136 ARIC participants with eGFR < 60 mL/min/1.73 m^2^, discrimination for HF and HFrEF models in ARIC was good: 14-year HF model 0.747 (0.707, 0.787); 4-year HF *C*-statistic (95% CI) 0.776 (0.724, 0.829); HFrEF 0.737 (0.673, 0.802). For HFpEF, discrimination was lower but acceptable: 0.678 (0.613, 0.743). Calibration in ARIC was best for short-term HF, but worse for the long-term models. We evaluated the original PCP-HF and the PCP-HF refit to CRIC in the ARIC validation cohort. The refit PCP-HF had somewhat lower *C*-statistics than the original PCP-HF; for 14-year HF, these were 0.610 (0.546, 0.675) and 0.664 (0.574, 0.755). *C*-statistics for the protein models surpassed the refit PCP-HF for all HF outcomes, and surpassed the original PCP-HF for 14- and 4-year HF (see Supplementary data online, *Table S3*).

#### Time-dependent AUC and subgroup analyses

**Figure 4:**
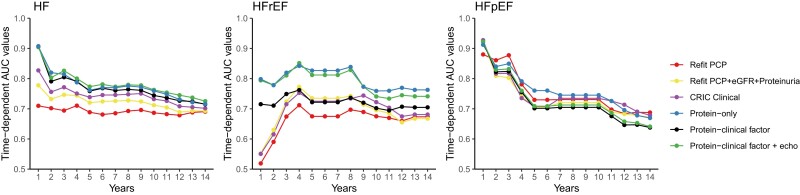
Time-dependent area under the curve. Time-dependent AUCs are shown for each risk model in the 20% testing set in CRIC (*n* = 479 participants)

To capture differences in discrimination at different time horizons, we evaluated annual time-dependent area under the curves (AUCs) for clinical, protein, and hybrid models for all 14-year outcomes. For HFpEF in particular, discrimination of clinical and protein models was better in the short-term (≤4 years) (*Figure 4*). We examined the discrimination of the protein models in subgroups of age, gender, race, DM, CVD, and eGFR and found the models performed similarly in subgroups (see Supplementary data online, *Figure S2*).

## Discussion

We have performed a large-scale proteomic analysis of HF in CRIC, a CKD cohort with in-depth phenotyping of participants and careful adjudication of HF events. We analysed 4638 unique plasma proteins in 2906 participants of CRIC and validated our findings in 1136 participants from ARIC with CKD. We identified over 200 proteins associated with incident HF after adjustment for eGFR at Bonferroni statistical significance (46 for HFpEF and 26 for HFrEF). Pathway analyses highlighted the importance of ECM biology, fibrosis, and inflammation. We identified six proteins with potentially causal associations, and four are currently druggable targets (FCG2B, IGFBP3, CAH6, ASGR1).^28^ Applying machine learning, we developed and validated proteomic risk models for HF, and protein models for short- and long-term HF, and for HFrEF, that surpass the extensively validated PCP-HF model^39,40^ among patients with CKD (*Structured Graphical Abstract*).

### Biological insights

Among individual proteins that are associated with HF in CRIC and validated in ARIC, many have roles in ECM remodelling. It is notable that many proteins have roles in the vascular ECM, and could reflect the biology underlying a causative association between HTN (itself caused by or causing CKD) and HF. SVEP1, an ECM protein found in vascular smooth muscle, was associated with incident HF and HFpEF in our study; it has previously been linked to atherosclerosis,^13^ and has been shown to predict hospitalization or death in patients with prevalent HFrEF.^41^ We also identified several less well-known ECM proteins. MFAP4 is an ECM protein manufactured in vascular smooth muscle cells related to fibrinogen and is a marker of pulmonary and hepatic fibrosis.^42^ Animal studies have shown that ROR2 is up-regulated in cardiac fibroblasts in remodelling hearts.^24^ FBLN5 is a secreted ECM protein found in large arteries^26^ necessary for the production of elastin polymers and modulates microfibril networks.^43^ FBLN5 knockout mice have ventricular diastolic dysfunction.^27^ We also noted an association for the ECM protein EGF-containing fibulin-like extracellular matrix protein 1,^17,18^ which has been shown previously to associate with HF.^44^ It is remarkable that no protein–HF associations had an interaction with EF < 50% at the statistical significance threshold that accounted for multiple testing. Visualization of HRs for HFpEF and HFrEF by scatterplot also suggests that associations of individual proteins for these two outcomes are similar. There is ample evidence of common pathophysiology between HFpEF and HFrEF, such as fibrosis, endothelial dysfunction, and inflammation.^45^ Furthermore, patients with HFpEF may have left ventricular systolic dysfunction when their functional reserve is tested.^46^

Consistent with the biology of individual proteins, the GO terms represented in our study were enriched with proteins relevant to the ECM. For example, within the category ‘positive regulation of synapse assembly’, we find thrombospondin-2, an ECM protein that induces cardiac remodelling by activating matrix metalloproteases, collagen production, and transforming growth factor-β.^35^ In the family labelled ‘proteoglycan biosynthetic process’, we find insulin-like growth factor I, which influences cardiac contractility, metabolism, hypertrophy, apoptosis^36,37^ as well as other members of the ECM including glycosaminoglycan xylosylkinase and carbohydrate sulfotransferases 9, 11, and 12. The family of ‘cell adhesion’ proteins includes many ECM proteins: cadherin 8 facilitates vascular smooth muscle cell–ECM interaction in cell models,^38^ and bone sialoprotein 2 complexes with hydroxyapatite, to form mineralized matrix^47^ (*Table 3*). The pathway ranked highest in Ingenuity Pathway Analysis was hepatic fibrosis, which is comprised of ECM proteins (see Supplementary data online, *Table S10*). Non-alcoholic fatty liver disease^48^ and hepatic fibrosis^49^ are common among individuals with HF. Additional pathways are important to uraemic cardiomyopathy, including those relevant to myocardial energy metabolism.^50^ We note that in the GSEA analysis, the insulin receptor signalling pathway via phosphatidylinositol 3-kinase was represented at *P* < .001 for overall HF (see Supplementary data online, *Table S9*).

The proteins identified by MR as potentially causal factors of incident HF have established or plausible roles in atherosclerosis and inflammation. FCG2B, IGFBP3, CAH6, and ASGR1 are druggable targets.^28^ FCG2B is a receptor for the Fc region of IgG; it modulates antibody complex processing by B cells, and its function has been researched in the setting of viral infections, malignancy, and autoimmune disease.^51,52^ FCG2B is the target of Xmab5871, a drug in phase 1 and 2 trials for autoimmune disease.^28^ IGFBP3 is a carrier for insulin-like growth factor 1 (IGF1). IGF1 is protective against atherosclerosis,^53^ and deletion of IGF1 receptors in mice results in dilated cardiomyopathy.^54^ IGFBP3 is the target for salvianolic acid, a drug being investigated in clinical trial for vascular dementia.^28^ CAH is implicated in vascular calcification.^55^ The well-known diuretic acetazolamide inhibits CAH4 in the proximal renal tubule, and carbonic anhydrase is also found in the heart, vasculature, and lungs.^56^ Several new diuretics, and also drugs for treating bacterial infection via CAH, are in development.^28^ ASGR1 facilitates the endocytosis of plasma glycoproteins; genetic loss of function has been associated with reduced levels of LDL cholesterol and lower risk of coronary artery disease.^57^ It is a target for the drug AMG 529, which is in a phase 1 trial for CVD. MSRE on macrophage foam cells facilitates endocytosis of LDL and accumulation of cholesterol in arterial walls. FCRL4 inhibits B-cell receptor signalling, and could potentially protect against myocardial inflammation.^58^

### Risk models

The PCP-HF clinical model was derived in community-based cohorts with low prevalence of CKD and relatively low risk of CVD, in contrast to CRIC participants who all have CKD and many have CVD comorbidities. Variables in PCP-HF include age, gender, DM, current smoking, SBP, hypertension treatment, total cholesterol, HDL, BMI, and QRS duration.^7^ Clinical models that we developed in this study for HF and HF subtypes in CRIC included age, DM, SBP, BMI, and QRS interval. They differed from the refit PCP-HF by including CVD comorbidities such as MI, stroke, and atrial fibrillation, as well as eGFR, proteinuria, and haemoglobin. Associations of clinical factors and all HF outcomes are listed in Supplementary data online, *Table S2*.

#### Protein model for the primary outcome (long-term heart failure)

For the primary outcome of 14-year HF in CRIC, the 48-protein model for HF [*C*-statistic (95% CI)] of 0.790 (0.735, 0.844) in the 20% testing set surpassed both the original PCP-HF (*P* = .001) and refit PCP-HF (*P* < .001). Evaluation of the protein models and original PCP-HF model for the outcomes of 4-year and 14-year HF, within subgroups of gender and race, demonstrate superior discrimination of the protein model in all subgroups (see Supplementary data online, *Table S11*). It is important to note that this subgroup analysis required evaluation of the protein models’ performance in a portion of participants that were included in the development of the protein model, and thus could overestimate the discrimination of protein model. Taken together, comparisons of protein models to PCP-HF (either refit or original) and to clinical models that include echocardiographic features suggest that proteins can substitute for clinical factors, without detriment to the model’s discrimination. Presumably, proteins encode the information for clinical and echocardiographic measures. Moreover, in each multi-protein model, roughly 25% of the proteins are potentially druggable targets; risk stratification based on modifiable risk factors may be used to monitor responses to lifestyle or pharmacological interventions.

#### Protein models for the secondary outcomes (short-term heart failure, heart failure with preserved ejection fraction, and heart failure with reduced ejection fraction)

The 30-protein model for the secondary outcome, 4-year HF, had significantly better discrimination than the original PCP-HF (*P* = .002), refit PCP-HF or CRIC clinical model (*P* < .05 for latter two). Given that the proteome varies over time, proteomics might provide the greatest advantage over clinical risk scores in the short-term. Additionally, shorter term risk models could help clinicians to identify individuals at high risk and implement intensive management strategies. Short-term prognosis is especially important for patients with shorter life expectancy, as is the case for many patients with CKD and multiple comorbidities.

The 33-protein model for HFpEF had nearly equivalent discrimination to the original PCP-HF (0.772 [0.695, 0.849]), but somewhat better discrimination than the refit PCP-HF (0.781 [0.696, 0.865] vs. 0.735 [0.635, 0.835]). Nine proteins in the protein model are druggable targets. The HFpEF risk model could potentially be developed into a modifiable risk score, used to monitor a patient’s risk after lifestyle or medical intervention and enrich the event rate in future clinical trials. It is notable that while 33 proteins were selected into the HFpEF model, only five proteins were selected into the HFrEF model, and in both CRIC and ARIC, the five-protein model had significantly better discrimination than the original or refit PCP-HF.

While our goal was to create informative, biologically-based risk models that could be further developed for clinical use, we did evaluate metrics of specificity, PPV, and NPV for 14-year and 4-year HF (see Supplementary data online, *Table S18*). For both refit PCP-HF and multi-protein models, when sensitivity is 80%, the models are expected to have excellent NPV, moderate specificity, and modest PPV. This reflects the known property of PPV, e.g. that it is directly related to disease incidence and tends to be lower if the incidence is lower. This suggests that these models would be useful for identifying low risk patients who could be managed conservatively, and for those at high risk, additional testing and close monitoring might be warranted.

### Limitations

Our study has numerous strengths, but we also acknowledge limitations. Notably, our MR analysis provides valuable leads to proteins causally associated with study outcomes that will need further validation in focused experimental and clinical investigations. While the CRIC population is well-phenotyped and permits extensive multivariable adjustment, any unmeasured confounders may bias the assessments of individual proteins as independent risk markers. We excluded patients with kidney failure on dialysis from the analyses, and censored participants who started dialysis, thus our results may not generalize to patients with kidney failure on dialysis. Additionally, our results may not generalize to patients with CKD at very young or old ages, high or low extremes of BMI, no history of HTN or treatment for HTN, or with autoimmune kidney disease (infrequent in CRIC) or polycystic kidney disease (excluded from CRIC). We have not tested our risk models in cohorts without CKD, so we do not yet know the degree to which our findings are specific to CKD. Our validation cohort for proteomic findings in ARIC differed somewhat by age, eGFR, and event rate compared to CRIC, and while this validation serves to establish our proteomics models as generalizable, more external validation is needed. Ideally, we would have conducted MR in one sample within a cohort of participants with CKD, but CRIC sample size was not adequate to support one-sample MR. We measured circulating and not tissue proteins. Future studies using alternate approaches may be useful for tissue localization.

## Conclusions

In conclusion, we present the largest proteomic study of incident HF and HF subtypes in participants with CKD to date with more than 13 million individual protein measurements, in a well-phenotyped population of nearly 3000 participants. Our analyses reveal multiple novel individual protein risk factors for HF, and we show that multi-protein risk models have better discrimination for incident HF than the PCP-HF. Druggable targets within our multi-protein risk models and significant MR findings may provide much needed information and impetus for developing new therapeutics.

## Supplementary data

Supplementary data are available at *European Heart Journal* online.

## Supplementary Material

ehae288_Supplementary_Data

## Data Availability

The CRIC data are available from the CRIC Study group upon request and with a Data Use Agreement. Data requests can made be by contacting the CRIC Scientific and Data Coordinating Center at cri-projmgmt@lists.upenn.edu.

## Pre-registered Clinical Trial Number

None supplied.
