# Supplementary material for: Incident heart failure in chronic kidney disease: proteomics informs biology and risk stratification
Source: Eur Heart J. 2024 May 17;45(30):2752–67. doi: 10.1093/eurheartj/ehae288 (PMC11313584; doi:10.1093/eurheartj/ehae288)
Supplement: ehae288_Supplementary_Data [file ehae288_supplementary_data.zip › EHJ_Revised Supplemental Methods_040924.docx]

**Supplemental Methods**

Participants: The Chronic Renal Insufficiency Cohort (CRIC) study was designed to investigate risk factors for the progression of CKD, cardiovascular disease, and overall mortality in persons with CKD.^1^ The CRIC study was approved by the Institutional Review Boards of the participating centers and the research was conducted in accordance with the principles of the Declaration of Helsinki. All study participants provided written informed consent. For clinical risk modeling, the number of participants was 2131 for incident HF, 2021 for incident HFrEF and 2288 for incident HFpEF (depending on numbers of available patients with required clinical factors).

Protein Quality Control: We included 59 proteins (1.2% of included proteins) with CVs between 20-50%, and CVs for individual proteins associated with HF are listed in **Supplemental Table 1.** The median intra-assay CVs, from plasma of healthy individuals are reported as ≤5%.^2, 3^ We conducted our own quality control study using samples from CRIC and also from patients attending a renal clinic. Median split duplicate CVs were ≤5% and median within-subject variability over a median interval of 1 week was <9%.^4^

CRIC Clinical factors Information on demographics and clinical history was obtained by self-report through questionnaires administered at the CRIC parent study baseline visit (Visit 3). Diabetes was defined as documented medical history, current or previous use of diabetic medications, or elevated fasting blood glucose. Blood pressure was averaged over three measurements performed in a standardized fashion in a seated position at rest using a calibrated sphygmomanometer. Analytes such as lipids, creatinine and phosphate were measured at the CRIC Central Laboratory at the University of Pennsylvania. Samples for cystatin C were processed using a Siemens BNII Nephelometer at the CRIC Central Laboratory, with an intra–assay coefficient of variation of 4.9%. Serum creatinine through 2008 was measured by an enzymatic assay from Ortho Clinical Diagnostics and thereafter using the Jaffe method from Beckman Coulter; creatinine measures were all subsequently normalized to an isotopic standard derived from mass spectrometry.^5^ Proteinuria was quantified by 24 hour urine collection.

Among the clinical and laboratory factors at Visit 5 (current study baseline) that we considered as predictors or covariates, there were <7% missing data for all, except for treatment of hypertension (HTN) (18% missing), proteinuria (8% missing), parathyroid hormone (PTH) (27.5% missing), NTpro-BNP (100% missing for visit 5), left ventricular mass index (LVMI) (20% missing), and ejection fraction (EF) (14.5% missing). We used Visit 3 proteinuria for 205 participants. We checked the correlations of the SomaScanV4 aptamer measures of PTH (Spearman correlation 0.89 with Visit 5 PTH measures) and NT pro-BNP (correlation 0.69 with NT-proBNP measures at baseline) and decided to use aptamer measures for these two markers that were available in all participants. We imputed missing data for baseline LVMI and EF using the R program  impute.rfsrc. eGFR was calculated using the validated CRIC equation that includes age, gender, race, cystatin C and creatinine.^6^

HF adjudication: Given the challenges of adjudicating HF for patients who have initiated dialysis, follow-up was censored at onset of kidney failure (N=707 patients who progressed to kidney failure were censored). Each HF adjudication was performed by two physicians, who reviewed hospital records and then came to a consensus on whether the hospitalization was definite or probable HF. CRIC criteria for HF include hospital notes, exam findings, chest X-ray, cardiac biomarkers and cardiac imaging when available, with details published previously.^7^ From among 390 incident HF events, 252 adjudication forms included estimation of EF with magnetic resonance imaging or echocardiography at the time of hospitalization. For participants whose EF was not quantified during the hospitalization, we used a CRIC research echo performed within 1 year before (N= 36) or 1 year after (N =14), unless the patient had a documented MI between imaging and hospitalization. Preserved EF was defined as EF≥50%. HFrEF or HFpEF were distinguished in 302 out of 390 HF events.

Individual protein associations with HF outcomes

The Cox proportional hazards model was used to assess the association between individual proteins and the primary (14-year HF) and secondary outcomes (4-year HF, 14-year HFrEF, and 14-year HFpEF.) In each instance, we constructed models with three levels of adjustment: (i) no adjustment, (ii) adjustment for eGFR only, or (iii) full adjustment for age, race, gender, DM, HTN, SBP, BMI, eGFR, UPCR, HDL, LDL, NT-proBNP, PTH, serum albumin, and hemoglobin. We chose to include eGFR in all models because of the direct effect of eGFR for increasing low molecular weight proteins, causing higher circulating levels of these proteins. For this reason, we adjusted for eGFR to increase the likelihood that markers we identify are mediators of CVD, rather than being solely eGFR markers. Bonferroni corrected significance levels were reported after adjusting for 4638 comparisons among proteins resulting in a significance level of p<1.0 x 10^-5^. The Benjamini-Hochberg (BH) method was used to control the false discovery rate (FDR) at 5%.^8, 9^

We explored whether protein associations differed between HFpEF vs. HFrEF (EF<50%) by selecting proteins associated with 14-year HF at a false discovery rate (FDR)<0.05, and visualizing effect sizes by scatterplot and formal interaction testing.

Risk Models

Clinical models: We examined the following clinical models for HF: 1) Original PCP-HF with published coefficients in four subgroups of gender and race;^7^ 2) PCP-HF, refit to CRIC; 3) refit PCP-HF + eGFR + proteinuria; and 4) a novel clinical HF model developed in the CRIC population. Since the original PCP-HF was not developed in a CKD population, refitting its coefficients in CRIC could yield improved discrimination compared to the original. For the refit PCP-HF, the following clinical variables were refit to the CRIC cohort: age, gender, diabetes (DM), fasting glucose, DM treatment, current smoking, SBP, HTN treatment, total cholesterol, HDL, BMI, QRS interval. We added race as a variable, rather than present race and gender-specific equations as was published for the PCP-HF, to facilitate comparisons within one 20% testing test. Next, we added eGFR and proteinuria to the refit PCP-HF.

We evaluated predictive performance by calculating Harrell’s C-index^10^ or Receiver Operating Characteristics Area under the Curve (AUC) in the testing set.^10^ We evaluated model calibration in the training set with calibration bar plots to visualize agreement between predicted and observed risk in quintile or quartile groups of participants defined by predicted risk. We further conducted stability analyses of our elastic net(EN) models to ensure that results were not overly dependent on the specific training / test set partition. This involved repeating the entire EN procedure on five alternate random partitions into training and test sets.

*Additional analyses for protein risk models:* For each protein model, we calculated time-dependent AUC values annually for years 1 to year 15 using the testing set data. We examined the discrimination of the 14-year protein models in subgroups of age, gender, race, DM, CVD, and eGFR. Sensitivity, specificity, positive predictive value (PPV) and negative predictive value(NPV) were calculated for 14-year and 4-year models.

Functional enrichment

For a given protein that was measured by two or more aptamers, the aptamer measurement with the largest effect size was used in the analysis. For a given aptamer that was annotated by multiple UniProt identifiers, the first identifier was used.^10-12^ Our primary method of enrichment analysis was over-representation analysis (ORA) using Gene Ontology (GO). GO biological process terms were ranked using Hypergeometric distribution with statistical correction as implemented in the R package, clusterProfiler.^13^ Using ORA, we examined proteins associated with each of three outcomes after adjustment for eGFR, at FDR<0.05, for 14-year HF (846 proteins), HFrEF (88 proteins), and HFpEF (160 proteins), relative to the background of all proteins measured by SomaScan. As a secondary enrichment analysis method provided by the same R package, we applied gene set enrichment analysis (GSEA) to rank relevant GO biological process terms. For GSEA, we produced a ranked list of all measured proteins using the formula: -log10(p) * sign(HR-1), where HR is the hazard ratio and p is the p-value per protein. Ingenuity Pathway Analysis (IPA) was utilized to examine canonical pathways, as we have described previously.^10-12, 14^ The Fisher right-tailed exact test was used to calculate a p value to determine the probability that the association of the differently expressed proteins in the measured data set, and the pathway is explained by chance alone. Ingenuity Pathway Analysis was utilized as an alternate pathway analysis tool of canonical pathways.

Mendelian Randomization

We used a two-sample Mendelian Randomization (MR) analysis to investigate the potential causal effects of 62 proteins associated with HF in CRIC, in analyses adjusted for eGFR at FDR<0.05. The list of 62 proteins included proteins that validated in ARIC and proteins included in risk models for 14-year HF, HFrEF or HFpEF. We obtained protein quantitative loci (pQTLs) from summary data in the deCODE database^1^ (<https://www.decode.com/summarydata/>), which includes genome-wide association studies (GWAS) of plasma protein levels measured with Somascan V4 in 35,559 Icelanders. We restricted the instrumental variants to *cis*-pQTLs using a Bonferroni p value threshold of 1.8×10^-9^. *Cis*-variant was defined as within one megabase (Mb) upstream or downstream of the transcription start site of the corresponding protein-coding gene. 41 proteins with *cis* pQTL in deCODE were retained for MR analysis. We performed LD clumping (r^2^ < 0.1) to prune the correlated pQTLs using the European population reference panel and R package TwoSampleMR^3^. For proteins with one SNP selected after clumping, we estimated the causal effect by the Wald ratio test. If more than one SNP were selected, we applied the inverse variance weighting method for multi-SNPs. We searched for associations of each IV with HF outcomes in summary data of the Heart Failure Molecular Epidemiology for Therapeutic Targets (HERMES) Consortium (<https://cvd.hugeamo.org>), a GWAS comprising 47,309 cases and 930,014 controls of European ancestry across 26 studies^4^. The statistical threshold for MR results was FDR<0.2; this corresponds to raw p-value of 0.05. Analyses were performed with R package MendelianRandomization.^2^ deCODE contributed 380,930 participants’ data to HERMES, but the 35,559 deCODE participants that were included in pQTL analyses comprise only 3.6% of HERMES, an overlap unlikely to significantly bias our results.^15^

External Validation

We performed external validation for individual proteins and for protein-only risk models in 1163 participants from the Atherosclerosis Risk in Communities (ARIC) Study^16^ with CKD defined by eGFR<60ml/min/1.73m^2^ who had the SOMAscan V4.0 measures performed on plasma samples at ARIC Visit 5. Reduced ejection fraction in ARIC was defined as ≤ 50%, and clinical adjudication has been described previously.^17^ We performed validation of 20 individual proteins with the highest and 10 proteins with the lowest HRs for 14-year HF, 14-year HFrEF and 14-year HFpEF after adjustment for eGFR. The criterion for validation was a p <(0.05/30 = 0.0017), based on 30 proteins carried forward for each outcome. We examined discrimination and calibration of protein risk models for 4-year and 14-year HF, 14-year HFrEF and 14-year HFpEF, after adjustment for differences in baseline hazard, but retaining coefficients developed in CRIC. ARIC follow-up time was 4 years for short-term HF model, and 8.58 years for long-term models. Analyses were performed using R version 4.2.2.

**References**

1. Feldman HI, Appel LJ, Chertow GM, Cifelli D, Cizman B, Daugirdas J, Fink JC, Franklin-Becker ED, Go AS, Hamm LL, He J, Hostetter T, Hsu CY, Jamerson K, Joffe M, Kusek JW, Landis JR, Lash JP, Miller ER, Mohler ER, 3rd, Muntner P, Ojo AO, Rahman M, Townsend RR, Wright JT and Chronic Renal Insufficiency Cohort Study I. The Chronic Renal Insufficiency Cohort (CRIC) Study: Design and Methods. *J Am Soc Nephrol*. 2003;14:S148-53.

2. Candia J, Cheung F, Kotliarov Y, Fantoni G, Sellers B, Griesman T, Huang J, Stuccio S, Zingone A, Ryan BM, Tsang JS and Biancotto A. Assessment of Variability in the SOMAscan Assay. *Sci Rep*. 2017;7:14248.

3. Niewczas MA, Pavkov ME, Skupien J, Smiles A, Md Dom ZI, Wilson JM, Park J, Nair V, Schlafly A, Saulnier PJ, Satake E, Simeone CA, Shah H, Qiu C, Looker HC, Fiorina P, Ware CF, Sun JK, Doria A, Kretzler M, Susztak K, Duffin KL, Nelson RG and Krolewski AS. A signature of circulating inflammatory proteins and development of end-stage renal disease in diabetes. *Nat Med*. 2019;25:805-813.

4. Dubin RF, Deo R, Ren Y, Lee H, Shou H, Feldman H, Kimmel P, Waikar SS, Rhee EP, Tin A, Chen J, Coresh J, Go AS, Kelly T, Rao PS, Chen TK, Segal MR and Ganz P. Analytical and Biological Variability of a Commercial Modified Aptamer Assay in Plasma Samples of Patients with Chronic Kidney Disease. *J Appl Lab Med*. 2023.

5. Joffe M, Hsu CY, Feldman HI, Weir M, Landis JR, Hamm LL and Chronic Renal Insufficiency Cohort Study G. Variability of creatinine measurements in clinical laboratories: results from the CRIC study. *Am J Nephrol*. 2010;31:426-34.

6. Anderson AH, Yang W, Hsu CY, Joffe MM, Leonard MB, Xie D, Chen J, Greene T, Jaar BG, Kao P, Kusek JW, Landis JR, Lash JP, Townsend RR, Weir MR, Feldman HI and Investigators CS. Estimating GFR among participants in the Chronic Renal Insufficiency Cohort (CRIC) Study. *Am J Kidney Dis*. 2012;60:250-61.

7. Dubin RF, Deo R, Bansal N, Anderson AH, Yang P, Go AS, Keane M, Townsend R, Porter A, Budoff M, Malik S, He J, Rahman M, Wright J, Cappola T, Kallem R, Roy J, Sha D, Shlipak MG and Investigators CS. Associations of Conventional Echocardiographic Measures with Incident Heart Failure and Mortality: The Chronic Renal Insufficiency Cohort. *Clin J Am Soc Nephrol*. 2017;12:60-68.

8. Harrell FE, Jr., Califf RM, Pryor DB, Lee KL and Rosati RA. Evaluating the yield of medical tests. *JAMA*. 1982;247:2543-6.

9. Hochberg Y and Benjamini Y. More powerful procedures for multiple significance testing. *Stat Med*. 1990;9:811-8.

10. Ferrannini E, Murthy AC, Lee YH, Muscelli E, Weiss S, Ostroff RM, Sattar N, Williams SA and Ganz P. Mechanisms of Sodium-Glucose Cotransporter 2 Inhibition: Insights From Large-Scale Proteomics. *Diabetes Care*. 2020;43:2183-2189.

11. Williams SA, Murthy AC, DeLisle RK, Hyde C, Malarstig A, Ostroff R, Weiss SJ, Segal MR and Ganz P. Improving Assessment of Drug Safety Through Proteomics: Early Detection and Mechanistic Characterization of the Unforeseen Harmful Effects of Torcetrapib. *Circulation*. 2018;137:999-1010.

12. Yang J, Brody EN, Murthy AC, Mehler RE, Weiss SJ, DeLisle RK, Ostroff R, Williams SA and Ganz P. Impact of Kidney Function on the Blood Proteome and on Protein Cardiovascular Risk Biomarkers in Patients With Stable Coronary Heart Disease. *J Am Heart Assoc*. 2020;9:e016463.

13. Yu G, Wang LG, Han Y and He QY. clusterProfiler: an R package for comparing biological themes among gene clusters. *OMICS*. 2012;16:284-7.

14. Walker KA CJ, Zhang J, Fornage M, Yang Y, Zhou L, Grams ME, Tin A, Daya N, Hoogeveen RC, Wu A, Sullivan KJ, Ganz P, Zeger SL, Gudmundsson EF, Emilsson V, Launer LJ, Jennings LL, Gudnason V, Chatterjee N, Gottesman RF, Mosley TH, Boerwinkle E, Ballantyne CM and Coresh J. Large-scale plasma proteomic analysis identifies proteins and pathways associated with dementia risk. *Nature Aging*. 2021;1:473-489.

15. Burgess S, Davies NM and Thompson SG. Bias due to participant overlap in two-sample Mendelian randomization. *Genet Epidemiol*. 2016;40:597-608.

16. The Atherosclerosis Risk in Communities (ARIC) Study: design and objectives. The ARIC investigators. *Am J Epidemiol*. 1989;129:687-702.

17. Zhao D, Guallar E, Vaidya D, Ndumele CE, Ouyang P, Post WS, Lima JA, Ying W, Kass DA, Hoogeveen RC, Shah SJ, Subramanya V and Michos ED. Cyclic Guanosine Monophosphate and Risk of Incident Heart Failure and Other Cardiovascular Events: the ARIC Study. *J Am Heart Assoc*. 2020;9:e013966.
